# Editorial: Regenerative surgery of the cornea

**DOI:** 10.3389/fmed.2022.1099876

**Published:** 2022-12-28

**Authors:** Jorge L. Alio

**Affiliations:** ^1^Division of Ophthalmology, Faculty of Medicine, Miguel Hernández University, Elche, Spain; ^2^Department of Research and Development, Vissum Miranza Alicante, Alicante, Spain

**Keywords:** corneal transplant, corneal regeneration, corneal transplant failure, corneal stromal regeneration, limbal epithelial transplantation, endothelial removal, keratoconus

Today we are seeing a continual growth in the volume of surgical corneal transplantations and the outcomes have indeed improved but still they are not completely satisfactory. It is estimated that in the European Union a number of around 30,000 corneal transplants being performed every year, with about 46,000 in the USA (Eye Bank Association of America, European Corneal Transplant Register, 2021). The numbers are very high but these grafts are affected in general by two types of potential hazards which are anatomical failure, which is a failure of the grafted tissue, or functional failure which is a failure of the visual outcome obtained with the grafting procedure. Both lead to unsatisfactory outcomes that are followed by new problems and in most cases a new corneal graft tissue is needed for a new transplant.

The causes of corneal transplant failure were well documented and studied by our research group multicentrical study in Spain (1). Anatomical failure in PKP is estimated to be between 20 and 30% at 5 years, in DALK about 20% and in DMEK, DSEK from 2 to 5%, with the main reasons being, primary graft failure, immunological rejection followed by primary graft failure, late endothelial failure, and negative impact of comorbidities such as glaucoma, ocular surface disease and reoccurrence of the original disease. The results were validated in Spain by a monocentrical study which confirmed that these issues were affecting also the outcomes of a high-volume center (2) and a general review of the topic published recently (3) proved that this is a worldwide problem. Functional outcomes failures happen frequently due to refractive reasons, inadequate corneal transparency, being estimated that in PRK and DALK over 30% of the successful anatomical cases end in best corrected visual acuity of < 0.4 (4). So, we may conclude that the outcomes of corneal graft surgery in 2022, even than far better than ever before, are still a problem and the consequences are that we need to be aware of the necessity to optimize anatomical and functional outcomes of modern corneal transplantation techniques. This can be accomplished by a better management of the main reasons for failure and indeed looking for new solutions based on modern approaches such as advanced therapies and stem cell corneal regeneration, avoiding tissue substitution.

At this regard, an important amount of investigation has been performed preclinically (5) but it was not until 2017 that the first pilot clinical trial on corneal stromal regeneration was performed in Lebanon under the direction of Jorge L. Alio. The outcomes of this initial experience demonstrate that this surgery is safe and the use of autologous mesenchymal stem cells either implanted alone (6), but also acellular human corneal laminas implanted in a corneal pocket with or without the impregnation of autologous stem cells lead to an important corneal bioactivation, increasing tissue and increasing some of the parameters of advanced cases of keratoconus (7–9). The evolution of the keratocytes in this environment is well documented and proves that autologous mesenchymal adipose derived stem cells transform into the phenotype of keratocytes (10). All these reports led to the clear perspective that we are facing a new approach for the treatment of corneal disease by corneal stromal regeneration (11).

Globally all of what has been evolving over the last year affects not only the stroma but also the ocular surface and the corneal endothelium (12).

In this Research Topic of Frontiers in Ophthalmology, El Zarif et al. report the studies on corneal stromal regeneration with a full review of the human clinical studies in the treatment of keratoconus. This paper summarizes everything that is now known about the use of isoplanar or curvilinear corneal lenticules in keratoconus but it is to be extrapolated to other corneal stromal dystrophies, as its positive impact in corneal transparency confirms previous investigations on animal models.

In the review on the regeneration of the corneal epithelium with limbal epithelial transplantation by Singh and Sangwan, the authors provide practical information aiming to provide practical guidelines for the use of limbal transplantation on a systematic basis on this difficult basis in which ocular surface reconstruction is necessary following corneal stem cell failure.

A very interesting report on the use of allogenic simple limbal epithelial transplantation from human leukocyte antigen-matched living related donor used to treat bilateral corneal chemical burns offers a fascinating technique to improve the ocular surface in these desperate cases in which very little is possible today with the available methods of corneal transplantation.

Finally, the outcomes of selective endothelial removal in a treatment of corneal endothelial dystrophy offers a perspective on the continuous evolution of corneal endothelial regeneration.

In summary, what in the future is going to happen in corneal regeneration surgery is already starting now. We are at the beginning of the emerging group of surgical techniques that target the avoidance of the use of human corneal tissue, improving the immunological outcomes, with more control, less negative induction of comorbidities such as glaucoma and, finally, better functional outcomes. I hope the reader of this special issue will be stimulated in the perception of this new perspective that modern cell biology, bioengineering and tissue regeneration techniques offer the modern corneal surgeon today.

## Author contributions

The author confirms being the sole contributor of this work and has approved it for publication.
